# Thermodynamic mechanism of the density and compressibility anomalies of water in the range − 30 < *T* (°C) < 100

**DOI:** 10.1038/s41598-022-05038-9

**Published:** 2022-01-24

**Authors:** Makoto Yasutomi

**Affiliations:** Department of Physics and Earth Sciences, Faculty of Science, University of the Ryukyus, Nishihara-Cho, Japan

**Keywords:** Condensed-matter physics, Structure of solids and liquids, Chemistry, Materials science, Mathematics and computing, Nanoscience and technology, Physics

## Abstract

Compared to normal liquids, water exhibits a variety of anomalous thermal behaviors. This fact has been known for centuries. However, the thermodynamic mechanisms behind them have not been elucidated despite the efforts of many researchers. Under such circumstances, the author theoretically reproduced the measured values of the density-temperature curve at 1 atm for water above 0 $^\circ$C. Then, the mystery of negative thermal expansion was clarified in relation to the shapes of the intermolecular interactions. In this paper, the author develops this line of work further and presents the interactions between water molecules to simultaneously reproduce the measured values of both the density-temperature curve and the isothermal compressibility-temperature curve in the range $-30<T(^\circ {\mathrm{C}})<100$ at 1 atm. Then, the thermodynamic mechanism that produces these thermal behaviors is clarified in relation to the shapes of the interactions between molecules. Unraveling the mystery of related phenomena in relation to the shapes of the interaction between molecules has been a traditional and fundamental method in physics since the days of Newton.

## Introduction

Newton constructed calculus and mechanics to establish a method for finding the motion of a mass point from the force acting on the mass point and the initial conditions. This set of Newtonian mechanics has been applied to planetary motion, and it has been shown that the data on planetary motion observed by Kepler can be reproduced, assuming that Newton’s universal gravitational force acts between the sun and the planet. Thus, it was discovered that Newton’s universal gravitational force acts between the masses of objects.

There is no direct theoretical way to know what functional form is taken by universal gravitation. Therefore, we first assumed several different universal gravitational functional forms. From among them, the form of the universal gravitational force $F=GmM/r^2$ was determined as the functional form that successfully reproduced Kepler’s observational data.

As the processing power of computers increased, Newtonian mechanics evolved into molecular dynamics. In this method, a substance consisting of innumerable molecules is approximated as a group consisting of a finite number of molecules. Then, based on Newtonian mechanics, the motion of each molecule is calculated using a computer by assuming the functional shape of the force acting between the molecules. Statistical mechanics techniques and the laws of thermodynamics can be used in this calculation to derive various thermodynamic quantities of matter.

Whether these thermodynamic quantities can reproduce the measured values for an actual substance is determined by the functional shape of the assumed intermolecular force.

It is known from thermodynamics and statistical mechanics that thermodynamic quantities in thermal equilibrium do not depend on the initial conditions of individual molecules or the path leading to the achievement of a thermal equilibrium state.

It is impossible to derive the interactions between water molecules from a basic equation such as the Schrödinger equation. At present, we can say that the functional form of an intermolecular interaction is determined as follows.

Several different types of water models have been proposed. One of them is the realistic water model, which was proposed with the main purpose of reproducing measured values with high accuracy (for example, TIP3P, TIP4P^1^, SPC/E^2^, TIP5P^3^, TIP4P/2005^4^, and mW^5^).

In TIP5P, for example, the oxygen atom of a water molecule is considered to be electrically neutral, and it is assumed that a Lennard-Jones potential acts between the oxygen atoms. Furthermore, the positive charge distribution and the negative charge distribution in the water molecule are represented by two positive point charges and two negative point charges, and it is assumed that a Coulomb force acts between the charges. The number of point charges and the values of those charges depend on the model. There are several parameters that characterize each water model, but their values are adjusted so that the results of the model calculations and the experimental results are in good agreement (see the above references or Yasutomi^6^).

However, even if the thermodynamic quantities obtained by this method reproduce the related phenomenon, it is not easy to discuss the thermodynamic mechanism behind the phenomenon in relation to the shapes of the intermolecular interactions.

The simplified model or core-softened model is a method proposed to solve this difficulty and elucidate the thermodynamic mechanism behind the given phenomenon in relation to the shapes of the intermolecular interactions.

In this method, the intermolecular interactions are approximated by simple functional shapes. Thermodynamic quantities can be calculated using the Monte Carlo method or the molecular dynamics method^7–19^.

Examples of water molecular interactions that have been proposed so far include the Lennard-Jones potential, Coulomb potential, van der Waals potential, Yukawa potential, and so on. In addition, interactions consisting of combinations of these potentials and various other forms of interactions have been proposed. However, it is not yet possible to reproduce the experimental results of water and elucidate the physics behind them.

A core-softened model for analytical calculation has been proposed in addition to numerical simulation. The self-consistent Ornstein-Zernike approximation (SCOZA) was studied by Betancourt-Cárdenas et al.^20–29^.

The SCOZA considers that intermolecular interactions consist of hard-core potentials and tail potentials. Various functional shapes have been proposed as tail potentials.

The author of this paper studied the thermodynamic properties of water, whose tail potentials consist of three or more Yukawa terms, using the SCOZA. He also showed that there are innumerable intermolecular interactions that can reproduce the measured values of the water density-temperature curve at 1 atm with high accuracy. He also presented many such interactions^26–28,30^.

The author went further and found the simplest interaction that could reproduce the measured values of water with high accuracy. Since the tail of this interaction consists of only two Yukawa terms, it is given by four parameters. However, one of them is used as a normalization constant, so there are only three free parameters. Therefore, it is not impossible to explore all of these parameters. In fact, the author applied this simplest interaction at 1 atm for water in the range $0<T(^\circ {\mathrm{C}})<100$. He found a number of interactions that could accurately reproduce the measured values of the water density-temperature curve^31^.

The core of the mystery of negative thermal expansion was explained in Yasutomi^32^ using simple and clear laws of thermodynamics. It is summarized here. For substances with positive thermal expansion, when the temperature is kept constant, the pressure *p* becomes a monotonically increasing function of the density $\rho$ on the pressure-density plane (see Fig. 2.2 of Yasutomi^32^). On this plane, the isotherms move to the high-pressure side as the temperature rises. The intersections of such a large number of different isotherms and a straight line with constant pressure $p_0$ (the red horizontal line in the previous figure) give a density-temperature curve (isobar) with respect to the pressure $p_0$.

From this isobar, it can be seen that the density decreases monotonically with increasing temperature. This is the simple and clear thermodynamic mechanism of positive thermal expansion. On this *p*–$\rho$ plane, if the density is kept constant, the pressure increases with temperature, so $\alpha = (\partial p/\partial kT)_\rho > 0$ (*T* denotes the absolute temperature and *k* is the Boltzmann constant). That is, $\alpha >0$ produces a positive thermal expansion.

From the above discussion, it is immediately clear that $\alpha <0$ produces a negative thermal expansion. That is, in a substance exhibiting negative thermal expansion, the isotherm moves to the low-pressure side in the opposite direction of the substance with positive thermal expansion as the temperature rises on the *p*–$\rho$ plane. The author actually elucidated this using a water model, using the laws of thermodynamics and the techniques of statistical mechanics, in relation to the shapes of intermolecular interactions (see Yasutomi^32^ and Yasutomi^27^).

A recent review^33^ describes simulations and experiments on anomalous liquid water, and several scenarios have been proposed to explain the anomalous properties. However, the thermodynamic mechanism that causes these changes has not yet been elucidated. Urbic and Dill^34^ state that “liquid water is considered poorly understood”.

In this paper, the author presents the simplest molecular interaction that simultaneously satisfies the measured values of both the isothermal compressibility-temperature curve and the density-temperature curve for liquid water in the range $-30<T(^\circ {\mathrm{C}})<100$ at 1 atm. Then, the mystery of the anomalous thermal behavior of isothermal compressibility is elucidated in relation to the shapes of intermolecular interactions. In addition, the difference between supercooled water and water at 0 $^\circ$C or higher is explained in relation to the shapes of intermolecular interactions.

Using his mechanics, during the process of theoretically reproducing Kepler’s observational data, Newton discovered that there is a universal gravitational force between the masses of objects. Similarly, in this study, it is indispensable to compare the measured and theoretical values of thermodynamic quantities for water. The author believes that theories that can reproduce experimental data should be regarded as capturing the essence of the phenomenon.

“Water model” section describes the adopted water model. “Method of calculation” section describes the calculation method. “Calculation results in the range 0 < *T* (°C) < 100” section compares the theoretical and experimental values in the temperature range $0<T (^\circ {\mathrm{C}}) <100$. “The reason for the minimum value of the isothermal compressibility occurring at approximately 46 °C” section discusses why the isothermal compressibility is minimal at 46 °C. To illustrate this phenomenon, virtual liquids VL1 and VL2 are introduced. “Calculation results in the range − 30 < *T* (°C) < 0” section compares the theoretical and experimental values in the temperature range of $-30<T(^\circ {\mathrm{C}})< 0$. “Summary and conclusions” section offers a summary and conclusions.

## Water model

Various thermodynamic quantities can be derived from excess internal energy $u(\rho , \beta )$. This energy is defined by the following equation.

1 $$\begin{aligned} u(\rho , \beta )=2\pi \rho ^2\int _0^\infty d r \ r^2 \phi (r) g(r). \end{aligned}$$

Here, $\beta =1/kT$, and *g*(*r*) is a distribution function. The thermodynamic potential is clearly related to the shape of the intermolecular interaction $\phi (r)$ when compared with other thermodynamic potentials. Therefore, it is more suitable to carry out this study with $u(\rho , \beta )$ than other thermodynamic potentials.

The quantity $u(\rho , \beta )$ can be obtained by solving the following equation using the SCOZA.

2 $$\begin{aligned} {\partial \over \partial \beta }\left( {1 \over {\chi _{\mathrm{red}}}}\right) =\rho {\partial ^2 u \over \partial \rho ^2}. \end{aligned}$$

Here, ${\chi _{\mathrm{red}}}$ denotes reduced isothermal compressibility and is defined by the following equation.

3 $$\begin{aligned} \left( {\partial \beta p \over \partial \rho }\right) _\beta ={\beta \over \kappa \rho }\equiv {1 \over {\chi _{\mathrm{red}}}}. \end{aligned}$$

Here, $\kappa$ is the isothermal compressibility.

It is assumed that the intermolecular interaction $\phi (r)$ in a certain thermal equilibrium state is composed of three parts: a hard-core potential, a soft-repulsive tail, and an attractive tail. This interaction is given by the following equation.

4 $$\begin{aligned} \phi (r)=\left\{ \begin{array}{l} \displaystyle {\infty , \qquad r<1}, \\ \displaystyle {-\sum _{n=2}^3 a_n{\exp [-z_n (r-1)] \over r}, \quad r\ge 1}, \end{array} \right. \end{aligned}$$

Here, $a_2$ is a normalization constant, and $a_3$, $z_2$, and $z_3$ are free parameters. Additionally, the hard-core diameter ${\sigma }$ is the unit of length, and the potential depth $\varepsilon$ is the unit of energy (see Yasutomi^35^ for details on the SCOZA used in this paper). To date, the author has dealt with potential tails consisting of three or more Yukawa terms^26–28,35^. In this case, except for the normalization constant, the potential tails contain 5 or more free parameters, so it is almost impossible to survey all such tails. However, since this interaction contains only three free parameters, it is not impossible to investigate almost all shapes. Therefore, it is very convenient to carry out this research.

## Method of calculation

If the thermal equilibrium state changes, the structure of the water molecule should change in addition to the changes in the coordination and orientation of the water molecule. Therefore, the interactions between water molecules should change constantly with changes in thermal equilibrium. The shapes of the interactions between water molecule changes with the temperature because the density is determined by specifying the temperature in the thermal equilibrium state at 1 atm. This is taken into account in this paper.

It has also been thermodynamically shown that the thermal equilibrium state does not depend on the path leading to its achievement. Therefore, at 1 atm, the shapes of the water molecule interactions at a certain temperature can be determined by the method described in the next subsection (see Yasutomi^35^ for the detailed calculation method of the SCOZA used in this paper).

### Calculation results in the range $0<T(^\circ {\mathrm{C}})<100$

Using the diameter of a rigid sphere $\sigma$ as the unit of length and the depth of the potential $\varepsilon$ as the unit of energy, the intermolecular interactions are made dimensionless. The intermolecular interactions are determined by four parameters, $a_2$, $a_3$, $z_2$, and $z_3$. If we focus on $z_2$ and $z_3$, there are innumerable combinations. Nevertheless, after several trial calculations, we find that $(z_2, z_3)$ = (34, 35) is a promising combination. Therefore, if one sets $a_2$ = 1 and lets $a_3$ be a free parameter, for water in the temperature range of $0<T(^\circ {\mathrm{C}}) <100$ at 1 atm, it is possible to obtain a series of intermolecular interactions that can simultaneously reproduce the measured $\rho$–*T* relation and $\kappa$–*T* relation.

As an example, the case $a_3/a_2$ = $-1.0034$ is described. First, for these four parameter values, the potential depth $\varepsilon$ is found. Dividing $a_2$ and $a_3$ by $\varepsilon$ gives the potential normalized by $\sigma$ and $\varepsilon$. Many isotherms can be obtained by starting from an infinite temperature and gradually lowering the temperature while keeping the shape of this normalized interaction constant.

Next, on the *p*–$\rho$ plane, the isobar ($\rho$–*T* relation) is found from the intersections of the straight line of $p=p_0$ (constant) and the above isotherms. Then, the density and the temperature are normalized by the maximum values of the density and the temperature, respectively, which yields the maximum density. Given that this normalized theoretical isobar and the experimental isobar match at the point with maximum density, the values of $p_0$ = 1.00 atm, $\sigma$ = 3.0743 Å and $\varepsilon$ = 2.077$\times$10$^ {-20}$ J are determined.

**Figure 1:**
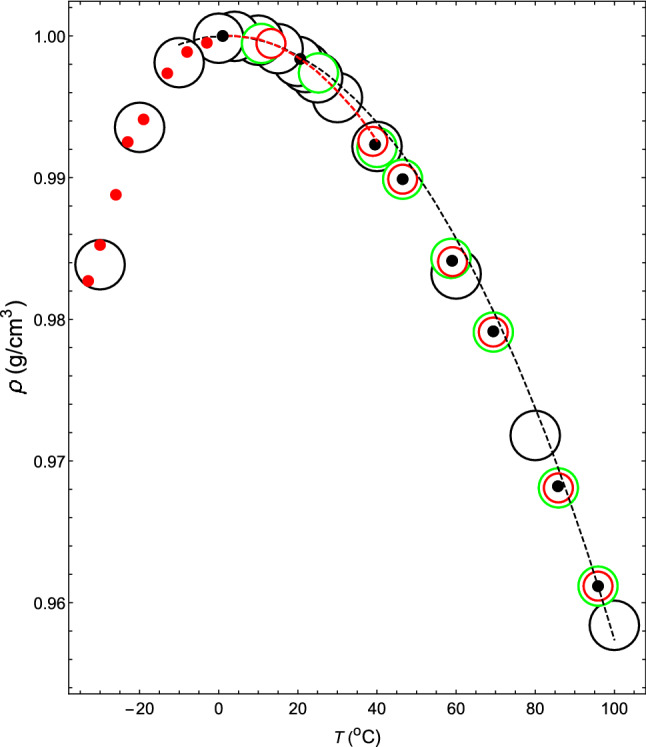
The $\rho$–*T* relation at 1.00 atm. Black dashed line shows the theoretical $\rho$–*T* relation obtained for ($z_2$, $z_3$, $a_2$, $a_3 / a_2$) = (34, 35, 108.615, $-1.0034$). Black open circles denote the measured values of water^4,36^. Black closed circles and red closed circles denote the theoretical $\rho$–*T* relation of water. Red dashed line shows the theoretical $\rho$–*T* relation obtained for ($z_2$, $z_3$, $a_2$, $a_3 / a_2$) = (34, 35, 111.685, $-1.0042$). Red open circles denote the calculated values of VL1 and green open circles denote the calculated values of VL2 (see “The reason for the minimum value of the isothermal compressibility occurring at approximately 46 $^\circ$C” for VL1 and VL2).

The theoretical isobar thus obtained is shown by the black dashed line in Fig. 1. Comparing the theoretical and experimental isobars (black open circles), they are, of course, consistent at 4 $^{\circ }$C. Although there is a slight discrepancy between the two as the temperature rises, they match again in the narrow temperature range around 95.88 $^{\circ }$C.

**Figure 2:**
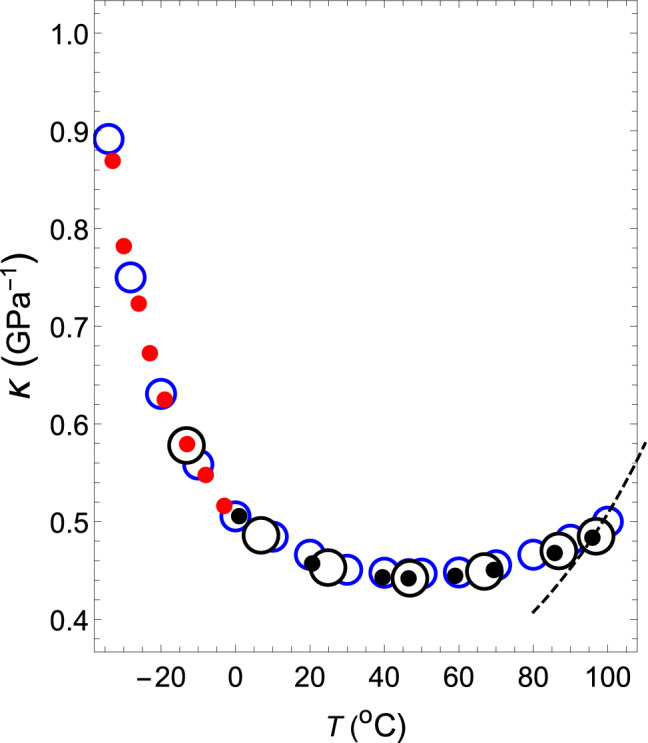
The $\kappa$–*T* relation of water at a pressure of 1.00 atm. The black dashed line shows the $\kappa$–*T* relation obtained for ($z_2$, $z_3$, $a_2$, $a_3 / a_2$) = (34, 35, 108.615, $-1.0034$). Black open circles: experimental values for water^37^. Blue open circles: experimental values for water^38^. Black and red closed circles denote theoretical values.

By changing the temperature of the theoretical isobar shown by the dashed line near the latter temperature, the theoretical $\kappa$–*T* relation at 1 atm can be obtained (the dashed line in Fig. 2) , which coincides with the measured relation at 95.88 $^{\circ }$C. Therefore, it is seen that the interparticle interaction given by ($z_2$, $z_3$, $a_2$, $a_3/a_2$) = (34, 35, 108.615, $-1.0034$) reproduces both the measured $\rho$–*T* relation and $\kappa$–*T* relation at 1 atm and 95.88 $^\circ$C with high accuracy.

**Figure 3:**
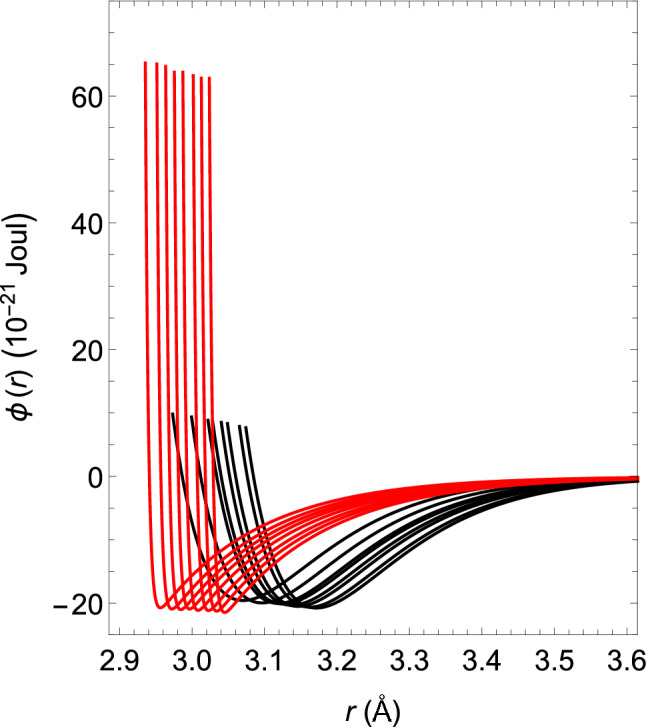
Temperature dependence of the functional shapes of intermolecular interactions. The solid black lines represent the functional shapes of water, VL1 and VL2. They have the same shapes regardless of the liquid type. However, their temperature dependence differs depending on the liquid type. If one lowers the temperature shown in Tables 1 and 2 in order from $T_1$ to $T_8$ (up to $T_7$ for VL1), the solid black line moves from right to left. The solid red line represents the temperature dependence of the functional shapes of the intermolecular interactions for supercooled water. When the temperature is lowered in the order shown in Table 3, the red line moves from right to left.

In exactly the same way, $(z_2, z_3)$ = (34, 35) can be kept fixed, and if one changes the value of $a_3/a_2$ in order to $-1.0035$, $-1.0037$, $-1.0038$, $-1.0039$, $-1.00398$, $-1.0042$ and $-1.00445$, the $\rho$–*T* relation and $\kappa$–*T* relation can be obtained, as shown by the black closed circles, in Figs. 1 and 2, respectively. The corresponding intermolecular interactions are shown by solid black lines in Fig. 3.

The theoretical values and experimental values match with high accuracy in the range $0<T (^\circ {\mathrm{C}})<100$ in Figs. 1 and 2.

### The reason for the minimum value of the isothermal compressibility occurring at approximately 46 $^\circ$C

In the case with normal substances, the isothermal compressibility decreases monotonically as the temperature drops. Additionally, as shown in Fig. 2, the isothermal compressibility of water decreases monotonically until approximately 46 $^\circ$C as the temperature drops. However, when the temperature is further lowered, it starts to increase. It has been a mystery for centuries as to why such strange behavior regarding isothermal compressibility occurs. In this subsection, we clarify this mystery in relation to the fact that the shapes of intermolecular interactions change with temperature.

When the water temperature $T_i$ ($^\circ$C) is lowered in order from $i=1$ to $i=8$, the shapes of the intermolecular interactions were shown in the previous section to move from right to left, as shown by the solid black line in Fig. 3. The value of $T_i$ is shown in Table 2.

**Figure 4:**
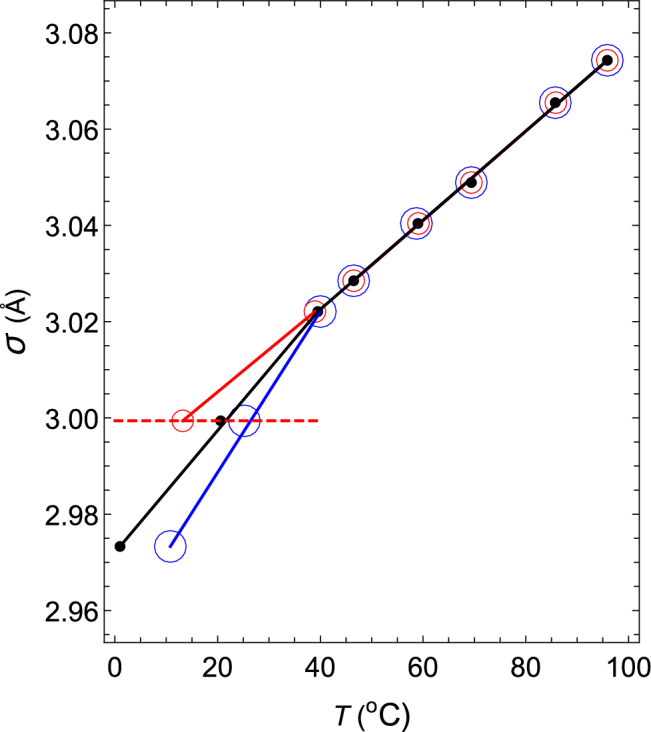
The changes in the hard-core diameters $\sigma$ of water, VL1 and VL2 with temperature. The diameters of water, VL1 and VL2 are shown in black, red and blue, respectively. The red dashed line denotes the $\sigma$–*T* relation obtained for ($z_2$, $z_3$, $a_2$, $a_3 / a_2$) = (34, 35, 111.685, $-1.0042$).

In addition, the change in the hard-core diameter $\sigma$ of water with temperature is shown in Fig. 4 with black closed circles. As shown in the figure, $\sigma$ is well approximated by two straight black lines with different gradients on the high-temperature side and the low-temperature side. The gradient changes rapidly around 46 $^\circ$C, where the isothermal compressibility $\kappa$ is the minimum value. It is thought that this change creates an anomalous temperature dependence for the isothermal compressibility. To confirm this, the following two virtual liquids are considered: VL1 and VL2. Then, the temperature change of the hard-core size is compared with that of water.

**Figure 5:**
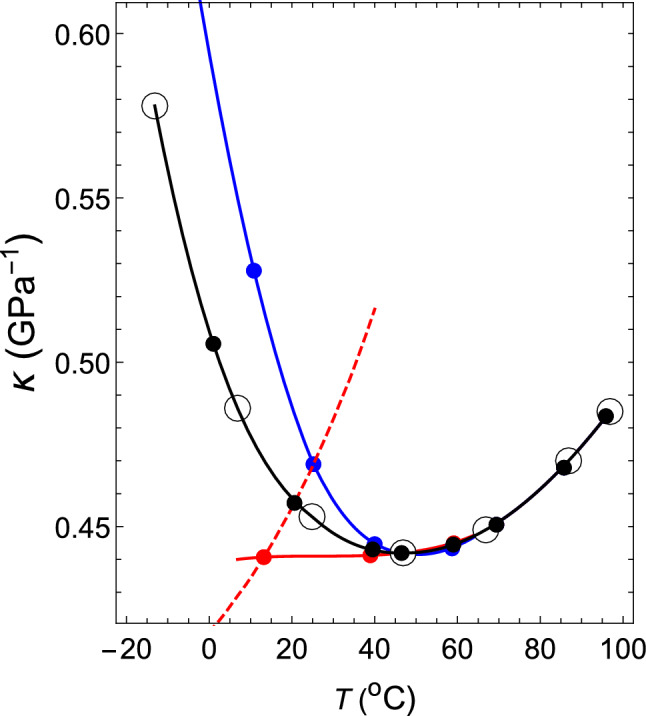
The $\kappa$–*T* relations at a pressure of 1.00 atm for water, VL1 and VL2. Black open circles: experimental values^37^. The red, black, and blue circles and lines are the $\kappa$–*T* relations for VL1, water, and VL2, respectively. The red dashed line shows the theoretical $\kappa$–*T* relation obtained for ($z_2$, $z_3$, $a_2$, $a_3 / a_2$) = (34, 35, 111.685, $-1.0042$).

In regard to the $\rho$–*T* relation, we assume that VL1 and VL2 exhibit the same relations as that of water. Regarding the $\kappa$–*T* relations of VL1 and VL2, both of them behave in the same way as water on the higher temperature side than 46 $^\circ$C. However, in the lower temperature region, $\kappa$ of VL1 decreases monotonically as the temperature decreases (red line in Fig. 5), but $\kappa$ of VL2 increases more rapidly than water (blue line) as the temperature decreases.

First, consider the case where ($z_2$, $z_3$, $a_2$, $a_3/a_2$) = (34, 35, 111.685, $-1.0042$). The intermolecular interaction given by these parameters has the shape shown by the 7th black solid line from the right in Fig. 3. The $\rho$–*T* relation obtained by fixing this interaction and changing the temperature at 1 atm is shown by a red dashed line in Fig. 1. This red dashed line is highly consistent with the experimental values of water in the temperature range of $0< T(^\circ {\mathrm{C}}) < 40$. The $\kappa$–*T* relation obtained by changing the temperature on this isobar is shown by a red dashed line in Fig. 5. This red dashed line indicates that $\kappa$ increases as the temperature rises. In addition, this red dashed line intersects the solid red line, solid black line, and solid blue line shown in Fig. 5 when the temperatures are 13.2, 20.6, and 25.2 $^\circ$C, respectively. Therefore, it can be seen that the temperature and the value of $\kappa$ at this intersection give the $\kappa$–*T* relations of VL1, water, and VL2. The same is true for $\sigma$ in the Fig. 4. The red circles, black circles, and blue circles on the red dashed line shown in Fig. 4 are the $\sigma$–*T* relations for VL1, water, and VL2, respectively. Therefore, it can be seen that this temperature dependence of $\sigma$ creates an anomalous temperature dependence for the isothermal compressibility of water. Additionally, the other closed red, black, and blue circles shown in Fig. 5 are obtained in exactly the same way (see Tables 1 and 2).

**Table 1:** The temperature dependence of the parameters that determine the intermolecular interactions of water, VL1 and VL2 at a pressure of *p* = 1.00 atm.

*T*	$a_2\,(\varepsilon )$	$a_3/a_2$	${\sigma }$ (Å)	$\varepsilon \,(10^{-20}{\mathrm{J}})$
$T_1$	108.615	− 1.0034	3.0743	2.077
$T_2$	108.994	− 1.0035	3.0655	2.0633
$T_3$	109.756	− 1.0037	3.0489	2.0509
$T_4$	110.139	− 1.0038	3.0404	2.0336
$T_5$	110.524	− 1.0039	3.0285	2.0138
$T_6$	110.832	− 1.00398	3.0221	2.0045
$T_7$	111.685	− 1.0042	2.9994	1.9967
$T_8$	112.661	− 1.00445	2.9733	1.9588

The value of each parameter is determined only by the value of temperature $T_i$ (*i* = 1–8), regardless of the liquid type.

However, the value of $T_i$ depends on the liquid type, as shown in the Table 2.

**Table 2:** Values of $T_i$ ($^{\circ }$C) ($i$ = 1–8) in Table 1 for water, VL1 and VL2.

Liquid type	$T_1$	$T_2$	$T_3$	$T_4$
VL1	95.88	85.85	69.4	59.1
Water	95.88	85.74	69.4	59.
VL2	95.88	85.74	69.4	58.7

Liquid type	$T_5$	$T_6$	$T_7$	$T_8$
VL1	46.49	38.95	13.2	–
Water	46.49	39.51	20.6	1.
VL2	46.49	40.	25.2	10.8

### Calculation results in the range $-30<T(^{\circ }{\mathrm{C}})<0$

It was mentioned in the previous section that a series of intermolecular interactions are obtained as a function of temperature in the range $0<T(^{\circ }{\mathrm{C}})<100$ by changing the values of $a_3/a_2$, as shown in Tables 1 and 2 with $(z_2, z_3)$ = (34, 35) fixed. The theoretical values of the $\rho$–*T* and $\kappa$–*T* relations obtained reproduce the experimental values with high accuracy.

However, if $(z_2, z_3)$ = (34, 35) remains fixed, the theoretical $\rho$–*T* relation for supercooled water below 0 $^{\circ }$C shows that the theoretical values gradually deviate from the experimental values as the temperature drops. Therefore, we must find other value combinations that are different from those mentioned above. However, after several trial calculations, if one fixes the combination of $(z_2, z_3)$ = (20, 700) and uses a series of interactions obtained by changing the values of $a_3/a_2$, it turns out that both experimental $\kappa$–*T* and $\rho$–*T* relations can be theoretically reproduced. The combination of these values, $(z_2, z_3)$ = (20, 700), is so different that it cannot be imagined from that above. The parameters and temperatures that determine these intermolecular interactions are shown in Table 3.

**Table 3:** Temperature dependence of parameters that determine the intermolecular interactions of supercooled water.

*T*	$a_2\,(\varepsilon )$	$a_3/a_2$	${\sigma }$ (Å)	$\varepsilon \,(10^{-20}{\mathrm{J}})$
$-3$	1.1936	− 3.45	3.0239	2.147
$-8$	1.1939	− 3.475	3.0129	2.126
$-13$	1.1941	− 3.5	3.0017	2.118
$-19$	1.1944	− 3.525	2.9874	2.114
$-23$	1.1946	− 3.55	2.9757	2.093
$-26$	1.1949	− 3.575	2.9637	2.102
$-30$	1.1952	− 3.6	2.9515	2.093
$-33$	1.1955	− 3.63	2.9356	2.074

The $\rho$–*T* relation and the $\kappa$–*T* relation are indicated in Figs. 1 and 2, respectively, with red closed circles. In each case, the theoretical results are highly consistent with the experimental values (the open black circles in Fig. 1 and open blue circles in Fig. 2, respectively).

**Figure 6:**
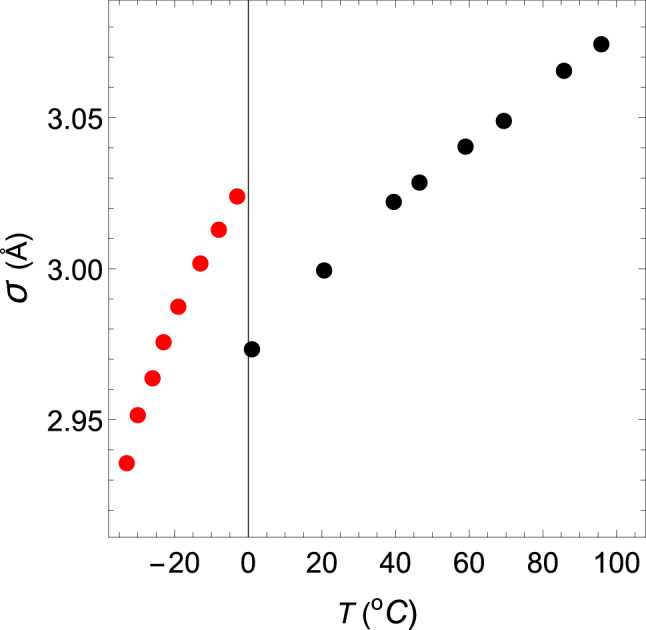
The $\sigma$–*T* relation of water.

The temperature dependence of the functional shapes of the intermolecular interactions is shown by the solid red line in Fig. 3. The functional shapes of the corresponding intermolecular interactions move from right to left in the order of decreasing temperature, as shown in Table 3. As the figure shows, the peak of the tail potential of supercooled water jumps discontinuously and dramatically around 0 $^\circ$C. Additionally, the $\sigma$–*T* relation of supercooled water is shown in Fig. 6 with red closed circles. It is also found that the value of $\sigma$ jumps discontinuously around 0 $^\circ$C.

Both the $\rho$–*T* relation and the $\kappa$–*T* relation change continuously near 0 $^\circ$C. However, the $\sigma$–*T* relation and the value of the tail potential at the hard-core contact change discontinuously near 0 $^\circ$C. This is an interesting finding in terms of understanding the difference between supercooled water and nonsupercooled water.

## Summary and conclusions

Thermodynamics and statistical mechanics show that the thermodynamic properties of matter are determined by the forces acting between the particles that make it up. Therefore, to fundamentally elucidate the mystery regarding the thermal behavior of matter, the topic should be discussed in relation to the shapes of particle-particle interactions. Such problem-solving methods can be said to be the most traditional and essential elucidation methods in physics since the days of Newton.

The author discussed the mystery of the negative thermal expansion of water in Yasutomi^35^ and Yasutomi^27^. It is said that it has long been known that $\alpha$ $=$ $(\partial p/\partial kT)_\rho$ $=$ $(1/kT)[u-\rho (\partial u/\partial \rho )+P]>0$ produces a positive thermal expansion and $\alpha <0$ produces a negative thermal expansion. The author showed that competition between *u*, $\rho (\partial u/\partial \rho )$ and *P* determines the sign of $\alpha$. He found a number of particle–particle interactions that change the sign of alpha at 4 $^\circ$C and elucidate the thermodynamic mechanism of negative thermal expansion in relation to the shapes of particle–particle interactions.

Several scenarios have been proposed^33^ to elucidate the thermodynamic mechanism of the density anomaly of liquid water. Some argue that the liquid–liquid phase transition is the cause of negative thermal expansion. However, they do not show how the liquid–liquid phase transition produces $\alpha <0$. The same is true for the other scenarios proposed to date. It is shown that the theory based on the core softened potentials can explain the water-like thermodynamic anomalies without the appeal of the liquid–liquid transition^8^. Liquid water is considered to be poorly understood^34^.

In this paper, we hypothesize that the intermolecular interactions in water are constantly changing depending on the state of thermal equilibrium. This is because, in addition to the coordination and orientation of water molecules, the structures of the molecules are also considered to be constantly changing depending on the thermal equilibrium state. We also present intermolecular interactions that reproduce experimentally obtained results regarding the $\rho$–*T* relation and $\kappa$–*T* relation for water with high accuracy in the range $-30<T(^\circ {\mathrm{C}}) <100$. As a result, it is clarified that the anomalous behavior of the isothermal compressibility of water is produced by the changes in the shapes of the intermolecular interactions or the changes in the diameters of the hard-core potentials with temperature.

Additionally, the maximum value of the tail of an intermolecular interaction (the value at the hard-core contact) or the diameter of the hard-core jumps up discontinuously at the melting point or freezing point of water. This is a surprising discovery, considering that the $\rho$–*T* curve and the $\kappa$–*T* curve are continuously changing at that point.

At present, it is impossible to derive the functional shapes of intermolecular interactions in water from basic equations such as the Schrödinger equation. The validity of the shapes must be judged by whether the assumed functional shapes can reproduce the measured values of some thermodynamic quantities of water. Newton’s law of universal gravitation was discovered in this way.

The interactions between water molecules used in this paper are composed of three parts: hard-core potentials, soft repulsive tails, and attractive tails. The latter two are each represented by one Yukawa term. There are four parameters that determine the functional form of an interaction, but since one is used as a normalization constant, there are only three free parameters. Therefore, it can be said that it is not practically impossible to explore all of these functional shapes.

The author does not mention the thermal behavior of supercooled water under − 30 $^\circ$C. Many anomalous thermal behaviors have also been found in this temperature range by experimentation or numerical simulations. Regarding the elucidation of the thermodynamic mechanism that generates such behaviors, the author believes that the method used in this paper will help elucidate them within the next few years. For that purpose, it is indispensable to compare detailed experimental values with theoretical values.

In this paper, the author presents intermolecular interactions that simultaneously reproduce the measured values of the $\rho$–*T* and $\kappa$–*T* relations of water. However, it should be considered that such interactions may exist in addition to those mentioned here. Nevertheless, even in that case, the author believes that the conclusions stated here will basically hold.

That said, SCOZA is not perfect. Therefore, the author believes that the conclusions drawn in this paper should be verified from various angles such as experiments and numerical simulations.

Finally, it is easy to imagine that the various anomalous thermal behaviors of water are thermodynamic phenomena created by the constant changes in the physical properties of water molecules themselves as the thermal equilibrium state changes.
